# Psychological issues in acquired facial trauma

**DOI:** 10.4103/0970-0358.73452

**Published:** 2010

**Authors:** Avinash De Sousa

**Affiliations:** Department of Psychiatry, Lokmanya Tilak Municipal Medical College and General Hospital, Mumbai, India

**Keywords:** Acquired facial trauma, psychological, face disfigurement

## Abstract

The face is a vital component of one’s personality and body image. There are a vast number of variables that influence recovery and rehabilitation from acquired facial trauma many of which are psychological in nature. The present paper presents the various psychological issues one comes across in facial trauma patients. These may range from body image issues to post-traumatic stress disorder symptoms accompanied by anxiety and depression. Issues related to facial and body image affecting social life and general quality of life are vital and the plastic surgeon should be aware of such issues and competent to deal with them in patients and families.

## INTRODUCTION

The face is often the seat of recognition for a human being and living with a change in the appearance of one’s face as a result of injury, disease, burns or trauma is always a challenging task. Various medical, personal, social and psychological variables influence the process of adaptation and it is often difficult predict the course of adaptation in many cases.[1] It is important that the plastic surgery treatment team be aware that the ultimate goal of their work, improving patient quality of life, is determined not only by their surgical skills but also by a range of social and psychological factors. The present review aims to highlight the various psychological issues in facial trauma and to encourage higher standards of care for patients with acquired facial disfigurement, in-cluding paying of attention to psychosocial rehabilitation.[2]

The psychological aspects in patients with acquired facial may not be completely addressed by the plastic surgery treatment team alone. The primary goal of plastic surgeons is to provide patients with the highest standards of surgical care and most members of the team have not been given adequate training to address psy-chosocial concerns. In addition there has not been enough research on the psychosocial responses and variables affecting the forms of acquired facial disfigurement.[3] The present review addresses one distinct form of facial disfigurement acquired during adulthood, i.e. acquired facial trauma and not at facial disfigurements through other causes like cancer or congenital anomalies. It is well know that psychological issues in response to acquired disfigurement are different and more pronounced than that to congenital craniofacial disfigurement. Patients with acquired facial trauma are likely to have some unique psychological characteristics.[4] We review the psycho-logical adaptation specific to facial trauma and discuss certain aspects of adaptation by individuals due to this ac-quired facial disfigurement, i.e. challenges in social functioning, body image issues, depression, anxiety and possible psychological morbidity.

## PSYCHOLOGICAL MORBIDITY OF PATIENTS WITH ACQUIRED FACIAL TRAUMA

The psychological needs of patients with acquired facial trauma are unique. It has been noted that patients with orofacial trauma were more likely to report symptoms of depression, anxiety, and hostility when compared to a matched normal control group for a period of up to 1 year post trauma.[5] Various studies have reported that 10-70% patients based on various factors may experience symptoms of depression and anxiety after a facial trauma.[6] This may be coupled with the fact that patients with acquired orofacial trauma have psychosocial problems like unem-ployment, lower education level and poor social support.[7] The symptoms of depression and anxiety in many cases may be sub-threshold and may not meet the full diagnostic criteria of a psychiatric disorder. This may often lead to diagnostic dilemmas, poor treatment of the problem and poor intervention. Reactions such as normative sad-ness, grief over the losses they have experienced, reactions to medications they may be taking and fatigue that results from treatment may be confused to being a depressive disorder or episode. Depression places the patient at increased risk for suicide, poor compliance with treatment and poor rehabilitation outcome. This in turn will affect the quality of life and recovery from the facial trauma.[8,9]

Depression and anxiety associated with facial trauma is often coupled with worries regarding recovery and length of the treatment process.[10] Facial trauma leads to disfigurement which also affects the social image of the patient.[11] Patients may express unhappiness regarding facial appearance after facial trauma and this may often led to social withdrawal and isolation. They may feel inferior to others in social presentation and may often feel a stigma associated with facial disfigurement.[12]

Often the injuries are due to family fights and interpersonal assault, while a third of the patients have a previous history of a facial trau-matic injury.[13] The recovery process after facial trauma is often lengthy and multiple surgeries with a multidisciplinary post-operative rehabilitation process may be needed. This may add to the frustration of the patient.[14] Injuries to key areas of the face like the eyes, ears and dental injuries often increase vulnerability to stress and impede recovery.[15] Significant difficulties in returning to premorbid levels of occupational functioning have been noted in these cases.[16]

Facial trauma pa-tients also report higher rates of somatoform symptoms, substance abuse, post-traumatic stress disorder symptoms, body image issues, stigmatization, lower quality of life and lower overall satisfaction with life.[17] They also report prob-lems in marital, occupational and social functioning.[18] They also report no nec-essary correlation between the degree of disfigurement and the type, extent and severity of psycholog-ical response.[19]

It is well known that facial trauma may occur in life-threatening situations and as a result of accidents or industrial mishaps.[20] This may often herald the onset of post-traumatic stress disorder (PTSD). The primary symptoms of PTSD include (i) re-experiencing of the trauma (e.g., having intrusive and distressing thoughts and/or distressing images and dreams); (ii) avoidance of thoughts, emotions or situations related to the trauma; and (iii) autonomic nervous system hyperarousal, including difficulties with sleeping, having an exaggerated startle response and experiencing increased irri-tability and tension.[21]

There have been studies documenting the evidence of PTSD symptoms in adult acquired facial trauma patients. It is also quite possible that a substantial portion of patients might experience sub-clini-cal forms of PTSD (i.e. not meeting the full diagnostic criteria) that can nevertheless substantially affect quality of life.[22] Individuals with acquired orofacial trauma who reported PTSD symptoms were more likely to also report pre-injury psychological problems, increased levels of stress and poor social support.[23] They are also likely to be elder, female and experience more injury-related pain.[24] Identification of PTSD symptoms can lead to further ex-ploration and uncovering of previously unrecognized additional psychological symptoms like depression and anxiety disorders.[25] It is well known that most psychological symptoms after facial trauma occur more in women as facial appearance and disfigurement concerns are more prevalent in them.[26]

## OTHER PSYCHOLOGICAL ISSUES IN ACQUIRED FACIAL TRAUMA

One study has compared plastic surgery patients undergoing treatment for facial cancer with patients undergoing reconstruction for scarring resulting from injury. Facial cancer patients reported lower levels of depression, anxiety, social concern and concern about their appearance as compared to the facial trauma patients.[27] Trauma-induced disabilities are often perceived to be random, unnecessary and unfair resulting in blaming and anger towards one’s self or others as well as being associated with idealizing one’s pre-injury physical appearance making the adjustment process more difficult.[28]

Additionally, facial trauma patients who have particular predisposing personality traits may be at increased risk for compromised quality of life [Table 1]. For example, higher levels of neuroticism were associated with lower quality of life and higher levels of alcohol consumption. Such problems would unquestionably complicate the process of psychologically adapting to a facial trauma, disfigurement and surgical reconstruction.[29]

**Table 1:** Premorbid personality factors that affect recovery from acquired facial trauma

Presence or absence of premorbid psychiatric illness[6]
Financial and social status[52]
Family history of psychiatric morbidity[53]
Family members involved in the same accidental event[20]
Family approach to the trauma and recovery[46]
General resilience of the individual[44]
Response to major life events in the past[54]
Survivor guilt-if present after the accident[55]
Accidental trauma versus an industrial or workplace trauma[56]
Compensation and litigation issues[57]

A wide range of variables are thought to influence psychological adjustment to acquired facial trauma. These include the nature of the patient’s so-cial support, presence of pre-existing psychological disorders and substance abuse problems and the extent of pain, as well as postoperative fatigue.[30] The use of passive styles of coping with the stress of the disease (e.g., through denial or avoidance) is more likely to result in more compromised quality of life.[31]

There are some other concerns that many patients with all forms of acquired facial disfigurement may have in common, that include chal-lenges in social functioning, body image adaptation and the possibility for psychological growth related to acquiring a facial disfigurement.[32] The goal of the plastic surgery team is to screen and determine if there are significant psychosocial problems affecting quality of life. If significant psychosocial problems are observed, the plastic surgery team ideally should provide appropriate feedback to the patient regarding how psychological factors can influence quality of life and be prepared to provide referrals to mental health professionals [Table 2].[33]

**Table 2:** Tell tale signs and symptoms when a referral to a psychiatrist is needed

Anger
Irritability
Poor social and family support
Crying spells
Loss of hope of recovery
Long in patient stay
Multiple surgeries
Depressed mood
Poor financial support
Loss of loved ones and family in the same traumatic event
Loss of job
Flashbacks and dreams regarding the event
Sleep problems
Chronic pain
History of psychiatric illness in the past
History of psychiatric illness in the family
Preoccupation with facial appearance after recovery

The greatest psychosocial challenge for most patients with any kind of facial disfig-urement is learning to cope with the social response to their facial appearance. For many individuals, these constant social challenges inevitably and eventually lead to social withdrawal. Many disfigured individuals narrowly limit their range of social interactions to immediate family members and to those social contacts re-quired for occupational functioning.[34,35] In its most extreme form, social withdrawal can result in what has been termed ‘social death’.[36,37]

Several extensive assessment tools have been designed to assess social functioning, including those specific to living with facial disfigurement particularly in the context of the plastic surgery consultation where the assessment of social functioning should be rather focoused.[38]

Individuals with an acquired facial disfigurement experience a long-term pro-cess of body image adaptation that significantly affects their quality of life. It has been found that facial trauma patients, as compared to control subjects, reported higher rates of negative body image thoughts and greater body image dysphoria in social situa-tions.[39] The body image concerns of disfigured people, like the body image concerns of most individuals, likely fall on a continuum from those having no psychological concerns to those patients who have an extreme level concern. Patients from the middle group are the vast majority of individuals who present for reconstructive surgery. They are able to function normally and yet still experience some body image experiences that affects their quality of life.[40]

There are some patients who despite having objective disfigurement resulting from acquired facial trauma report no significant concerns regarding their appearance.[41] This may be the result of psycho-logical denial and a lack of investment in their facial appearance. Some have an extreme level of body image concern that significantly affects their quality of life. Such patients are likely to experience a range of mental health problems that are clearly evident to the plastic surgery treatment team.[42]

There is now a compelling and ever-growing body of scientific literature documenting psychological growth in response to highly traumatic experiences.[43] A number of positive and negative coping styles, resilience factors and trauma interpretation methods determine recovery from trauma.[44] While more attention needs to be given to evaluating and treating the suffer-ing of patients with acquired facial disfigurement, it should also be kept in mind that some patients will demonstrate not only remarkable resilience, but also growth in the face of their experience.[45] A very important factor in recovery from any trauma is family support and culture. It is well known that the joint family system in India provides greater social support to the patient compared to the west where nuclear families prevail.[46] The cultural attitude towards illness, recovery, the sick role, work and disability are other factors that would help or impede this process.[47]

## PSYCHOLOGICAL PRINCIPLES FOR THE PLASTIC SURGEON

Some general principles for the psychologi-cal management of patients undergoing reconstructive surgery that are helpful must be kept in mind when handling such patients. The plastic surgery team needs to do whatever is necessary to allow the patients and families to create realistic expectations for the anticipated surgical outcome. The team should be as clear as possible regarding the length of time it will take to complete the reconstruction, the total number of surgeries and how much pain and life disruption will likely occur.[48]

One of the most important contributions that the treating surgeon can make to the care of patients is to take time to closely listen to their unique concerns and those of their family members about the surgery, its outcome and their experience living with disfigurement.[49]

Many patients require little extra psychological care other than just the routine attention given. They benefit from extra time, atten-tion and reassurance. The surgical team is clearly able, in most instances, to adequately respond to the needs of their patients.[50] However if it is felt that the patient and the family can be helped further by interacting with a psychiatrist, such a meeting should be facilitated.

There has been some progress made in addressing the specific psychosocial concerns of individuals with facial dis-figurement, including addressing the need for social skills development, learning how to adaptively cope with persistently negative social response, applying the particularly efficacious cognitive-behavioural forms of intervention to the specific concerns of those living with disfigurement and developing and disseminating effective psycho-educational materials.[51]

## CONCLUSION

It is important to emphasize that the most important ways in which the treatment team can enhance patients’ psychosocial rehabilitation is to be aware of the published clinical literature on the psychosocial adaptation of patients with acquired facial trauma. It is also important to routinely ask patients how they are cop-ing with the changes that have occurred since the change in their facial appearance and have an idea about the family’s view of this adaptation process.

The single most important step that plastic surgeons can make to ensure that their patients attain the highest level of psychosocial rehabilitation is to develop a consistent and trusting relationship with a mental health professional whom one can confidently and enthusiastically refer your patients.
